# New technique for culturing corneal epithelial cells of normal mice

**Published:** 2009-08-14

**Authors:** Takeshi Kobayashi, Ryuji Yoshioka, Atsushi Shiraishi, Yuichi Ohashi

**Affiliations:** 1 Department of Ophthalmology and Regenerative Medicine, Ehime University School of Medicine, Shitsukawa, Toon, Ehime, Japan; 2 Department of Ophthalmology, Ehime University School of Medicine, Shitsukawa, Toon, Ehime, Japan; 3 Department of Cell Growth and Tumor Regulation, Ehime University School of Medicine, Shitsukawa, Toon, Ehime, Japan

## Abstract

**Purpose:**

To describe a new method of culturing mouse corneal epithelial cells (MCECs).

**Methods:**

MCECs were isolated from C57/BL6 mouse corneas and cultured on type-I collagen-coated plastic dishes in low-calcium progenitor cell targeting medium (CnT-50). Expression of the mRNAs of N-terminal truncated isoform of p63 (*DNp63*), cytokeratin 12 (*K12*), and cytokeratin 14 (*K14*) were determined by reverse transcription-polymerase chain reaction (RT-PCR). To examine the differentiation capabilities, passage 3 (P3) MCECs at confluence were subcultured on amniotic membrane (AM) in a differentiation medium (CnT-30) until confluence. At confluence, 1 mM calcium was added and cultured for 4 more days. The expression of K12 in the stratified MCECs was analyzed by immunostaining.

**Results:**

The MCECs cultured in CnT-50 proliferated until at least P10. The number of cells at confluency at P3 was 61.8 (SD ±9.4, n=5) times that at P0. MCECs cultured on AM in CnT-30 with addition of calcium were stratified up to two to three layers, and the stratified MCECs expressed K12. *DNp63* mRNA was continuously expressed throughout the different passages, and *K12* mRNA was detected in P0 cells and the stratified MCECs on AM.

**Conclusions:**

Cultured MCECs maintain their proliferation and differentiation capabilities as well as their corneal epithelial cell characteristics. These results suggest that MCECs produced by this culturing method provide a useful experimental model which can enable further development of research of the corneal epithelium.

## Introduction

Mice are used in many different types of studies for several reasons: they are well-suited for genetic manipulations such as genomic sequence analyses; different strains with different characteristics are readily available, and many transgenic (Tg) and knockout strains have been created and are commercially available. Furthermore, in vitro approaches with cultured mouse cells allow the investigation of tissue or cell specific properties.

To investigate the physiological and pathological conditions of corneal epithelial cells, many corneal epithelial cell lines and primary culture systems have been established for humans and rabbits [1-6]. However, there have been few reports regarding the creation of a corneal epithelial cell line and primary culture system for mice.

Hazlett et al. [7] developed a method for short-term cultures of primary mouse corneal epithelial cells (MCECs), although they failed to subculture the cells past passage three, and the cultures may have been contaminated by fibroblasts. Since then, some researchers reported on the results of in vitro examinations of primary MCECs, and they were able to study these cells without any effect from adjacent tissue cells and matrices [8,9]. Unfortunately, a large number of eyes were used to obtain sufficient number of cells for the primary cultures, and the culture conditions were not stable among the different experimental groups.

Recently, Kawakita et al. [10] and Ma et al. [11] demonstrated that long-term cultures of MCECs could be achieved by culturing MCECs in keratinocyte serum-free medium. Although their technique required several weeks to establish a stable cell line and the probability of the establishment was 55%, there was a possibility that their method could establish a MCEC line. However, there was still some concern on whether the cells in this cell line maintained corneal properties, e.g., expression of ketratin 12.

Because it is important to have sufficient number of MCECs to perform reproducible experiments and to reduce the number of experimental animals used, it is necessary to develop an easily repeatable method to culture and grow MCECs that maintain the properties of normal MCECs. Thus, the purpose of this study was to develop a simple and reproducible method for culturing MCECs that will allow the cells to retain their proliferation and differentiation capabilities. To accomplish this, we used a low-calcium, low-bovine pituitary extract (BPE), serum-free progenitor cell targeted medium to culture MCECs.

## Methods

### Tissue preparation and cell culture

C57/BL6 mice (CLEA Japan Inc, Tokyo, Japan), aged 4-8 weeks, were handled in accordance with the guidelines in the ARVO Statement for the Use of Animals in Ophthalmic and Vision Research. Intact and viable MCEC sheets were prepared as described with some modifications [11]. In brief, the eyes were enucleated from the euthanized animals and incubated in DMEM/F12 (1:1 mixture; Invitrogen, Tokyo, Japan) containing 15 mg/ml dispase II (Roche Diagnostics, Basel, Switzerland), 100 mM sorbitol, and antibiotic-antimycotic (1X; Invitrogen) for 18 h at 4 ^°^C. The loosened corneal epithelial sheets were peeled off with forceps and incubated in 100 µl of 0.25% trypsin (Invitrogen) for 10 min at 37 ^°^C. To inhibit the activity of trypsin, 100 µl of 2 mg/ml soybean trypsin inhibitor (Roche Diagnostics) in PBS(-) was added to the medium, and the sheets were separated into single cells by pipetting. Then 2 ml of low-calcium, low- bovine pituitary extract (BPE), serum-free progenitor cell targeted medium (CnT-50; CELLnTEC, Bern, Switzerland) or low-calcium, serum- and BPE-free progenitor cell targeted medium (CnT-20; CELLnTEC) was added to the isolated cells. The cells were then transferred to type-I collagen coated 35 mm plastic dishes (Asahi Techno Glass, Funabashi, Japan). The cultures were incubated at 37^ °^C under 95% humidity and 5% CO_2_. The medium was changed every 2 to 3 days. Confluent cultures of MCECs were subcultured at a density 1×10^4^ cells/cm^2^.

### Preparation of cultured corneal epithelial cell sheets

To examine the differentiation potential of the MCECs, confluent cells at passage 3 (P3) were subcultured on amniotic membrane (AM) in low-calcium, serum- and BPE-free differentiation medium (CnT-30; CELLnTEC). When the cells had reached confluency, the medium (CnT-30) was changed to CnT-30 supplemented with calcium (1 mM) and the medium was changed daily for 4 days. The MCEC sheets on the AM were collected.

### RNA extraction and reverse transcription-polymerase chain reaction (RT-PCR)

For RNA extraction, the epithelial surface of normal 4-week-old C57/BL6 mouse corneas were scraped with a scalpel, and the epidermal layer was excised with scissors. Total RNA was extracted from normal mouse corneal epithelia (MCE) and normal mice epidermal (MED) tissue, and also from MCEC sheets, and MCECs harvested from confluent primary culture (P0) and P3 with RNeasy Mini kit (Qiagen, Tokyo, Japan). cDNAs were made by reverse transcription of the total RNA with the SuperScript VILO cDNA Synthesis Kit (Invitrogen) according to the manufacturer’s protocol.

PCR was performed with AmpliTaq Gold PCR Master Mix (Applied Biosystems, Tokyo, Japan) in 20 µl containing 2 µl of cDNA, 0.25 µM concentrations of primer, and 10 µl of the 2X PCR Master Mix. The mixture was subjected to 30 cycles of 15 s at 95 ^°^C for denaturation, 30 s at 60° C for primer annealing and extension. The PCR primers are listed in Table 1.

**Table 1:** PCR primers used in this study

**Target**	**Primer**	**Primer sequence**	**Amplicon length**	**GenBank accession**
K12	Forward	ACTAGAGCCGACCTGGAAGC	158	NM_010661
	Reverse	ACCTTGGTGAGATCCACTCC		
K14	Forward	ACCGCAAGGATGCTGAGGA*	103	NM_016958
	Reverse	GAAATCTCACTCTTGCCGCTCTG*		
DNp63	Forward	CTGGAAAACAATGCCCAGACTCA	126	AF075437 , AF075438, AF075439
	Reverse	TGCGTGGTCTGTGTTGTAGG		
ß-actin	Forward	CATCCGTAAAGACCTCTATGCCAAC*	171	NM_007393
	Reverse	ATGGAGCCACCGATCCACA*		

The asterisk indicates that the primer sequences for K14 and ß-actin were designed by Takara Bio Inc. (Otsu, Japan).

### Immunohistochemical analyses

MCEC sheets were fixed in 4% paraformaldehyde/phosphate-buffered saline (PBS) at 4 ^°^C. After fixation and dehydration in a graded series of ethanol, the samples were embedded in paraffin and cut into 5 µm sections. The sections were blocked in a solution containing 2% rabbit serum, 2% BSA, and 0.1% Triton-X 100 in PBS for 1 h, followed by incubation with 0.2 mg/ml of goat anti-cytokeratin 12 (Santa Cruz Biotechnology, Inc. Santa Cruz, CA) in CanGetSignal A (TOYOBO, Osaka, Japan) overnight at 4 ^°^C. Immunoreactivity to primary antibodies was made visible by secondary antibody conjugated with FITC (Vector Laboratories, Burlingame, CA). After rinsing in PBS, the sections were mounted on glass slides with ProLong Gold Antifade Reagent with DAPI (Invitrogen).

## Results

### Characteristics of cultured MCECs

**Figure 1:**
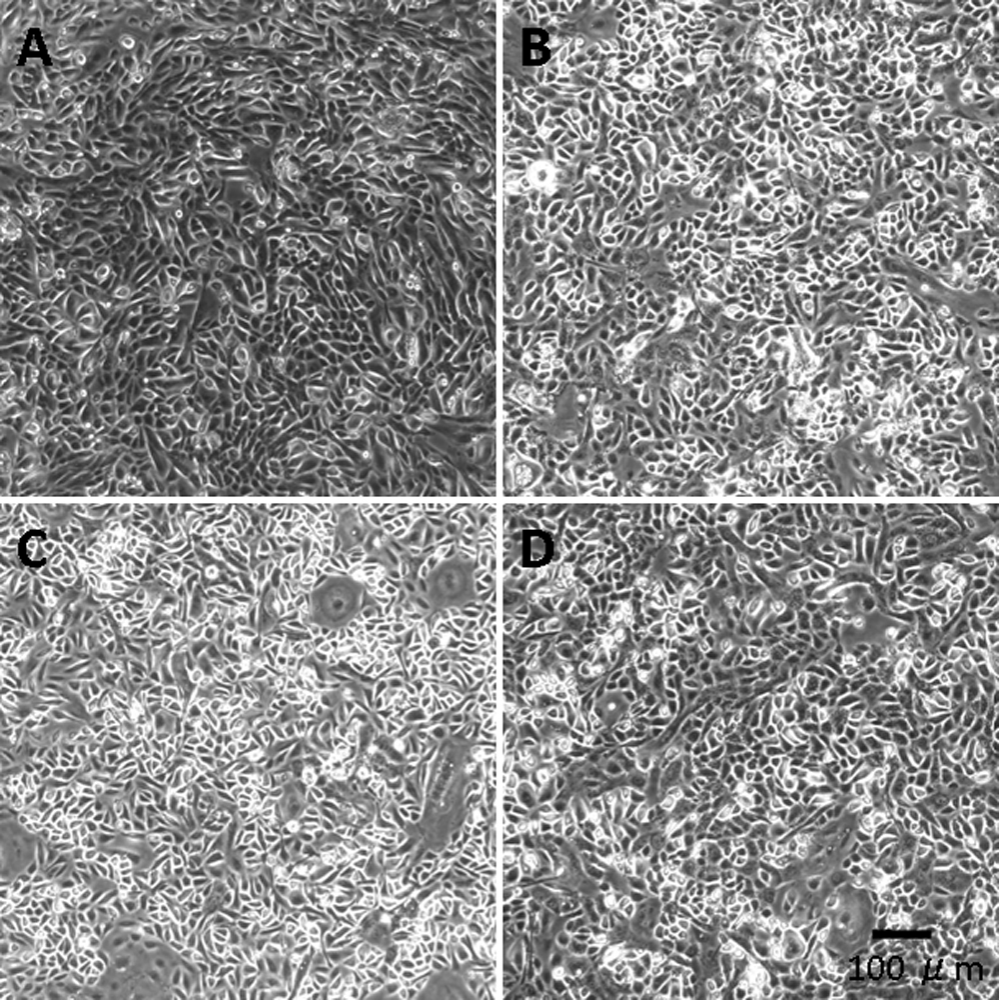
Phase contrast photomicrographs of mouse corneal epithelial cells (MCECs). MCECs at confluence in CnT-50 medium at different passages; **A**, passage 0 (P0); **B**, P3, and **C**, P10.  MCECs at confluency at P3 cultured in CnT20 (**D**). Scale bar; 100 μm.

Mouse corneal epithelial cells (MCECs), which were cultured on type-I collagen coated plastic dishes in low calcium, low BPE, serum-free medium (CnT-50), continued to proliferate until at least P10. The MCECs had a cobble stone-like appearance which did not change throughout the 10 passages (Figure 1A-C).

**Figure 2:**
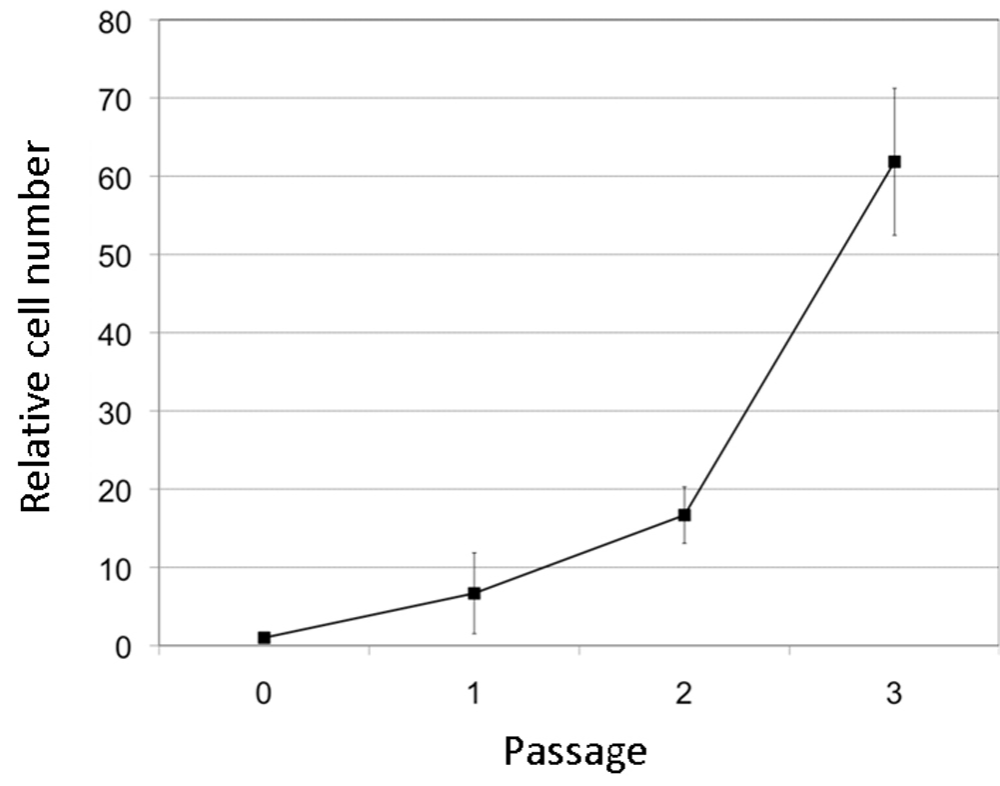
Growth of MCECs cultured in CnT-50. Each point is the mean of five individual experiments. The number of cells at confluency at each passage was counted and normalized to the number at P0. Error bar represents the standard deviations.

The MCECs in low-calcium, serum- and BPE-free medium (CnT-20) were also able to proliferation until at least P3 (Figure 1D), but the rate of proliferation of MCECs in CnT-20 was considerably slower than in CnT-50 (data not shown). The proliferation curve of MCECs in CnT-50 is shown in Figure 2, and each value was the mean of five experiments. The number of cells was counted at confluence of each passage, and was normalized to the value at P0. The means±standard deviations of cells at P1, P2, and P3 were 6.7±5.2 (n=5) times, 16.7±3.6 (n=5) times, and 61.8±9.4 (n=5) times that at P0. The interval from primary cell seeding to confluence of P3 ranged from 24 to 52 days.

### RT-PCR

**Figure 3:**
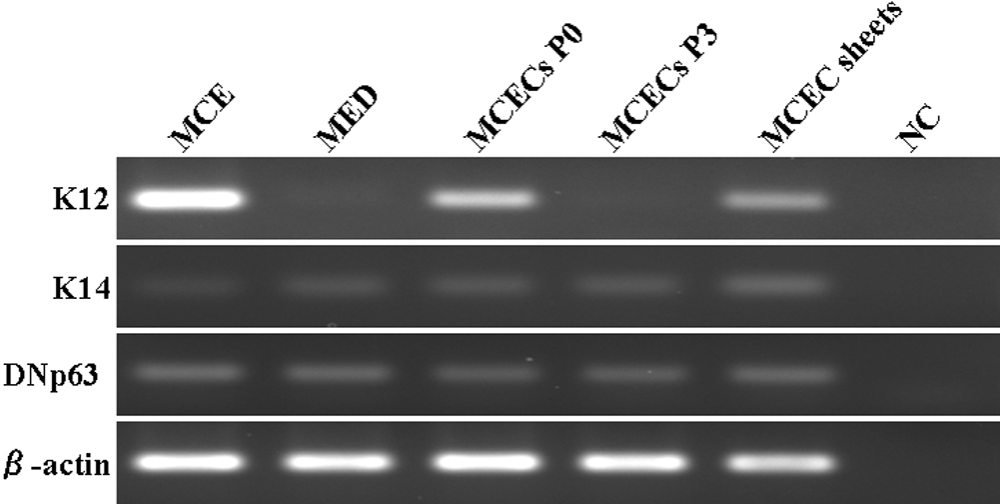
*K12*, *K14*, and *DNp63* mRNA expression.* K12*, *K14*, and *DNp63* mRNA expression in normal mouse corneal epithelia (MCE), normal mouse epidermis (MED), MCECs at confluence at P0 and P3, and MCEC sheets. PCR products were analyzed by 1.5% agarose gel electrophoresis. PCRs with no DNA template were used as negative control (NC).

*DNp63*, *K12*, and *K14* mRNA expression was detected in normal mouse cornea, and *DNp63* and *K14* but not *K12* were detected in normal mouse epidermis (Figure 3). The expression of *DNp63* and *K14* mRNA was detected in cells from P0 and P3 of MCECs and MCEC sheets, while the expression of *K12* mRNA was detected in MCECs at P0 and MCEC sheets but not at P3 (Figure 3).

### Differentiation potential of cultured MCEs

**Figure 4:**
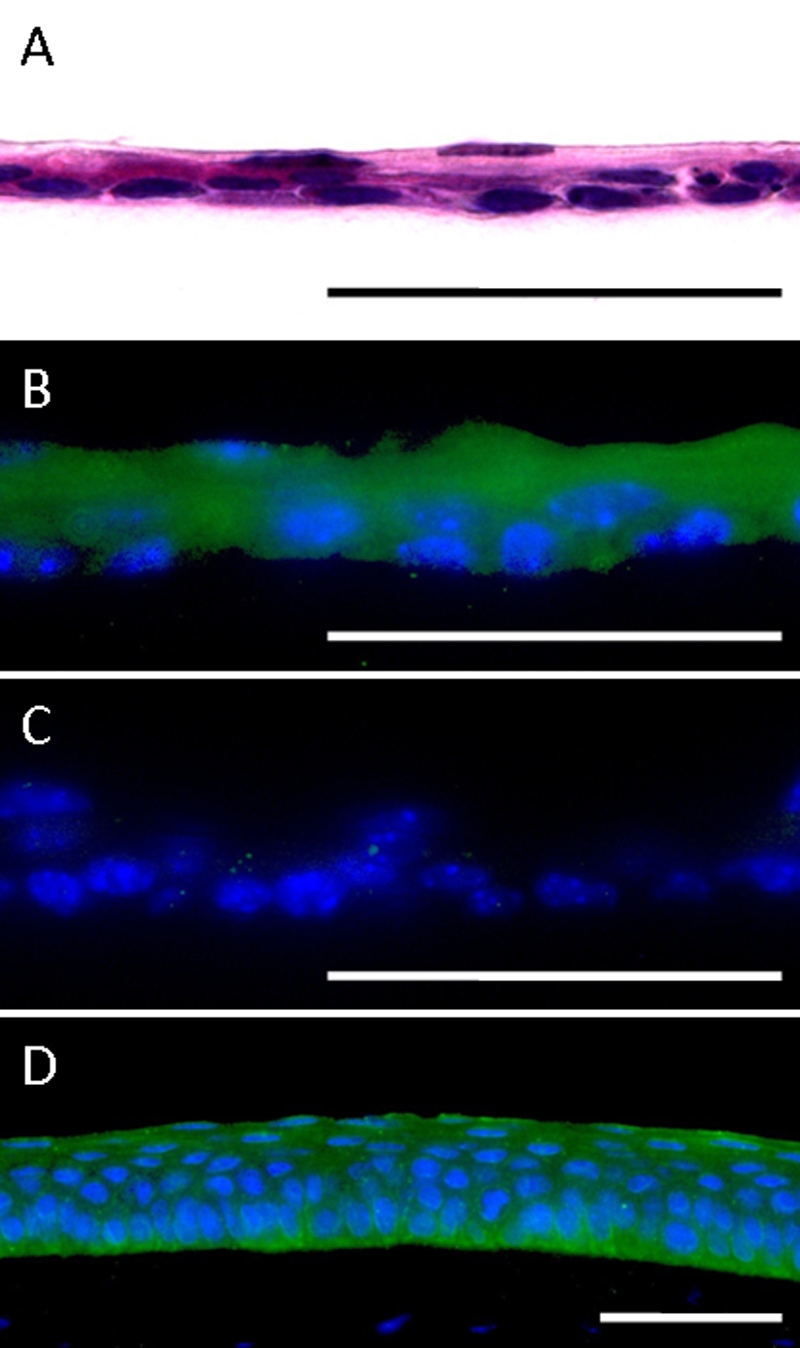
Immunohistochemical staining of MCEC sheets. Paraffin sections (5 µm) were stained with; **A** = H&E, **B** = immunostained with anti-cytokeratin 12, and **C** = normal goat IgG. Normal mouse cornea was immunostained with anti-cytokeratin 12 (**D**). Scale bar; 50 µm.

MCECs were subcultured on AM in serum- and BPE-free differentiation medium (CnT-30) supplemented with 1 mM of calcium. The cells proliferated and were stratified to two to three layers by four days (Figure 4A). Immunoreactivity to K12, a corneal epithelium specific differentiation marker, was detected in the stratified MCECs as seen in normal mouse corneal epithelia (Figure 4B,D). The MCEC sheets did not stain with nonspecific goat IgG (Figure 4C).

## Discussion

Our results demonstrated that a simple culture method can maintain MCECs up to P10. The MCECs were cultured on type-I collagen-coated plastic dishes in low-calcium serum-free medium, which is commercially available. The MCECs maintained their proliferation and differentiation capabilities, and had corneal epithelial cell characteristics.

Low-calcium, serum-free, or no BPE human corneal epithelial progenitor cell targeted (PCT) media (CnT-50 or CnT-20) have been used for isolation and proliferation studies of early passage human CECs [12,13]. Although the number of studies using these media has been limited, the PCT media appears to be effective for culturing human CECs. Because we were not aware of any studies using the PCT medium for MCECs, we have examined its effectiveness for MCECs. We found that the MCECs cultured in CnT-50 continued to proliferate until at least P10, and the morphology of the cells was not altered throughout the ten passages (Figure 1). A relatively faster rate of proliferation was observed in a medium containing low concentrations of BPE (CnT-50) than in a complete defined medium (CnT-20), although the morphology of the cells was not different (Figure 1).

Recently, Kawakita, et al. [11] and Ma, et al. [10] reported on methods for culturing MCECs, however, it required several weeks to establish a stable cell line of MCECs, and the probability of the establishing the cell line was 55%. On the other hand, we attempted to culture MCECs five times by our method, and were successful each time. Our analyses showed that the MCECs had proliferated to 61.8 times (ranged from 54.5 to 76.9) that at P0 within the three passages (Figure 2).

In the cultured MCECs, the expression of *DNp63* mRNA was detected throughout the passages examined (Figure 3). DNp63 is known to be a transcription factor that is essential for the differentiation of progenitor stratified squamous epithelial cells, e.g., corneal epithelial cells and epidermal cells [14,15]. During the differentiation of stratified squamous epithelial cells, DNp63 is expressed in the basal cells, but not in differentiated cells [14,15]. These results suggest that the differentiation capability was well maintained in MCECs cultured in CnT-50.

It has been recognized that multiple passaged cultured cells may alter their cellular properties. Indeed at P3, the MCECs cultured in CnT-50 did not express *K12* mRNA. Therefore, we next examined whether the MCECs maintain the corneal epithelial characteristics as well as the differentiation potential. Confluent P3 MCECs cultured on AM in differentiation medium (CnT-30) supplemented with 1 mM calcium expressed *K12* and were stratified up to two to three layers. Ma et al. [10] also demonstrated the differentiation potential and *K12* expression in cultured MCECs, however they failed to detect *K12* expression at the protein level. On the other hand, our immunohistochemical results clearly showed positive staining of K12 in the stratified MCECs grown on AM as seen in normal mouse cornea. It is well recognized that AMs provide a better microenvironment for corneal epithelial cells, and it has been reported that the use of AM promotes the corneal epithelial cell differentiation [16-18]. Thus, the expression of *K12* might be better with the AM, however MCECs cultured in CnT-50 still maintain the corneal epithelial characteristics after P3.

Our analyses also showed that the MCECs proliferated an average of 61.8 times more (range: 54.5 - 76.9) at P3 than at P0 (Figure 2). When cells were collected from ten mouse eyes and cultured to confluence in CnT-50, the number of cells reached 3.0×10^7^ by the third passage. This number is more than sufficient for most in vitro experiments.

In conclusion, a commercially available low-calcium, low-BPE, serum-free medium (CnT-50) made it possible to grow MCECs on type-I collagen coated plastic dishes. The relatively simple and reproducible results of culturing MCECs with CnT-50 should be valuable for laboratory experiments.
